# A cross-reactive plasmonic sensing array for drinking water assessment†‡

**DOI:** 10.1039/d3en00565h

**Published:** 2023-11-13

**Authors:** Justin R. Sperling, Baptiste Poursat, Laurie Savage, Iain Christie, Calum Cuthill, Badri L. Aekbote, Katie McGuire, Affar S. Karimullah, Jill Robbie, William T. Sloan, Caroline Gauchotte-Lindsay, William J. Peveler, Alasdair W. Clark

**Affiliations:** a James Watt School of Engineering, University of Glasgow Glasgow UK Alasdair.Clark@glasgow.ac.uk; b School of Chemistry, University of Glasgow Glasgow UK; c School of Law, University of Glasgow Glasgow UK

## Abstract

The continuous monitoring of remote drinking water purification systems is a global challenge with direct consequences for human and environmental health. Here, we utilise a “nano-tastebud” sensor comprised of eight chemically-tailored plasmonic metasurfaces, for testing the composition of drinking water. Through undertaking a full chemometric analysis of the water samples and likely contaminants we were able to optimise the sensor specification to create an array of suitable tastebuds. By generating a unique set of optical responses for each water sample, we show that the array-based sensor can differentiate between untreated influent and treated effluent water with over 95% accuracy in flow and can detect compositional changes in distributed modified tap water. Once fully developed, this system could be integrated into water treatment facilities and distribution systems to monitor for changes in water composition.

## Abstract 2

The continuous monitoring of remote drinking water purification systems is a global challenge with direct consequences for human and environmental health. Nanoplasmonic sensors can monitor treatment systems and warn of failures.

## Graphical abstract

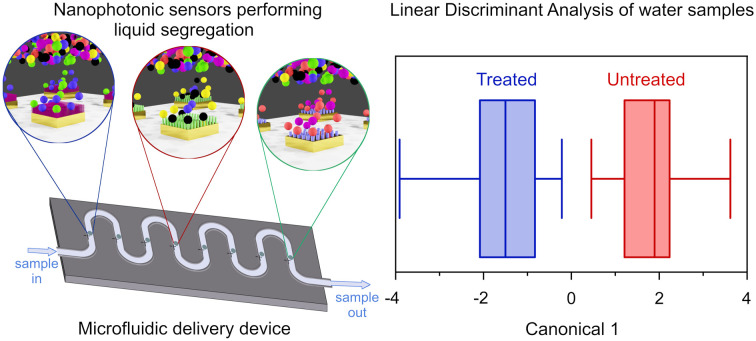

**Environmental significance**

The widespread challenge of monitoring decentralised drinking water purification systems and its impact on human and environmental well-being necessitates innovative solutions. In this study, we introduce a novel approach utilizing a “nano-tastebud” sensor consisting of chemically-tailored plasmonic metasurfaces. This ultrasensitive technology bridges the gap between nanoscale physics and practical applications, such as real-time water monitoring, crucial for safeguarding ecosystems and human health. By deciphering the optical response patterns generated by each water sample as it interacts with the sensor array, we can holistically detect variations in water composition across a library of samples from multiple different sites, and different treatment levels. The integration of this simple, robust technology into treatment systems may significantly improve early anomaly detection, profoundly impacting environmental preservation.

## 1. Introduction

Ensuring rapid detection of water composition changes in drinking water treatment works (DWTW) is of utmost importance to safeguard consumers. The most commonly employed method for detecting failures involves routine sampling and laboratory analysis to monitor parameters like pH, turbidity, conductivity, dissolved oxygen, organic carbon, nitrogen, and specific ions in the water.^1^ These parameters do not necessarily affect public health directly, but are important markers of water composition that can be used to indicate faults in water treatment processes. While analytical analyses are highly accurate at quantifying the molecular and ionic composition of sampled water, they provide only a snapshot of the system at the time of sampling. Considering that off-site laboratory analysis of samples from remote facilities can take several days to complete, there can be a significant delay between sampling and detection of significant changes in the system. Furthermore, limited access to laboratories in remote locations makes ‘regular’ and ‘routine’ monitoring difficult, if not practically impossible, as evidenced in the 2020 UN Sustainable Development Goals Report,^2^ which found that the quality of drinking water supplied to at least 3 billion people is unknown, due to lack of monitoring. To this end, there is a need for a new, low-cost, point-of-use monitoring system capable of rapidly identifying potential system failures.

To address the challenges of continuous water quality monitoring, a variety of sensing technologies have been demonstrated for on-site and inline sample analysis, including Raman spectroscopy,^3–6^ field-effect transistors,^7^ electrodes,^8,9^ wet-chemical microfluidic sensors,^10^ optical waveguide and fibre-based sensors,^11,12^ plasmonics,^4,13–15^ and off-the-shelf microcontroller-based sensors.^16^ While many of these technologies have high sensitivity and selectivity, each is only able to monitor a small range of components. This is beneficial in instances where a single pollutant or small range of components in the water are of key importance, but, given how complex both raw and treated water are, less useful as a holistic evaluation of water quality. In the European Union, for example, around 40 parameters, such as per- and polyfluoroalkyl substances (PFAS), nitrate/nitrite, and copper concentration are considered “essential” while 20 others, such as the colour, conductivity, ammonium, and manganese content are considered “proof of quality.”^17^ Additionally, this method of monitoring only accounts for the things we presume may be in the water, but does not typically account for “unknowns” that may also be present and are of interest for safety.^18^

Rather than build a device with multiple sensors designed to detect single specific components (many of which would be hard to analytically separate), we propose an optical tongue approach – a system of detection based on the principles of mammalian taste, whereby an array of cross-reactive sensors creates a sensing fingerprint for a given complex mixture, without needing to specifically target specific markers, and can measure subtle changes in that mixture thanks to holistic measurement of chemical differences.^19^ Cross-reactive sensing has shown utility in biomedicine,^20,21^ and for the detection of environmental pollutants^22^ and chemical threats.^23^ Here for the first time, we apply the principles of cross-reactive sensing in a nanoplasmonic, microfluidic device to monitor water composition.

We have previously demonstrated an array of chemically-tailored plasmonic metasurfaces (nano-tastebuds or NTBs), that act as an optical fingerprinting technology for whisky identification.^24^ By modifying each metasurface with a different self-assembled chemical monolayer (SAM) we can affect segregation of liquid samples at the sensing surface; different components within the sample segregating to each metasurface based on local supramolecular interactions. This, in turn, induces a change in the local refractive index experienced by the metasurface, measurable by a change in its plasmonic resonance wavelength. Since the surface chemistries are cross-reactive, we are able to ‘fingerprint’ complex mixtures based on statistical analysis of the combined resonance shifts.^24^ By building a library (or training set) of these optical ‘fingerprints’, it becomes possible to discriminate between and identify new, unknown samples without the need for prior knowledge of their contents.^20,25–27^

**Fig. 1:**
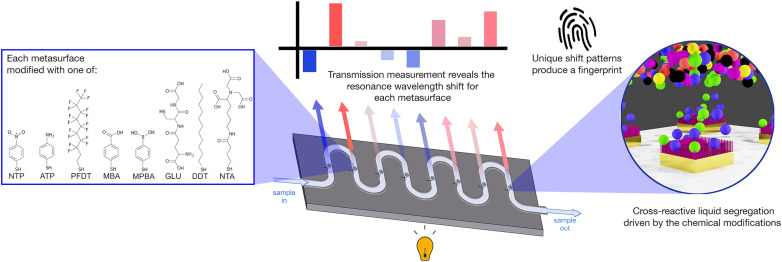
Schematic of the sensor concept. Eight metasurfaces are linked by a microfluidic channel. Each metasurface is modified with a different surface chemistry, providing partial-selectivity to the components in the water sample. Thus, each metasurface experiences a unique refractive-index environment, shifting its resonance wavelength value. By tracking the combination of resonance shifts we build a fingerprint for each sample.

We have now extended our optical tastebud approach to detect differences between water samples, as a water composition monitoring device. In this study, our device focused on differentiating between treated and untreated water at water treatment sites, as well as water at the end of distribution systems. We obtained raw influent (INF) water and treated effluent (EFF) water from various Scottish drinking water treatment works, as well as tap water samples from randomly selected consumer taps (all samples provided by Scottish Water). Organic carbon and ion content characterization of these water samples was used to guide sensor design (*i.e.* cross-reactivity choices). The sensor was integrated into a microfluidic enclosure that allowed each water sample to be tested in flow (Fig. 1). A pattern recognition map was generated that could successfully discriminate treated and untreated waters. This proof-of-concept shows the promise of this technology as a real-time sensor for the early detection of shifts in water composition; shifts that would enable prompt maintenance of a water treatment system, while subsequent lab-based analytical tests would pinpoint the underlying issue.

## 2. Experimental

### 2.1 Sample collection

Scottish Water provided 10 samples of influent (INF, I01–I10), 12 samples of effluent (EFF, E01–E12), and 3 sample of tap (TAP, T01–T03) water from various drinking water treatment works and randomly selected consumer taps across Scotland. Samples were aliquoted and frozen at −20 °C upon collection. Prior to analysis, samples were defrosted overnight at 4 °C and then filtered using a 0.22 μm PES membrane filter.

### 2.2 Inductively coupled plasma optical emission spectroscopy (ICP-OES)

Inductively coupled plasma optical emission spectroscopy (ICP-OES, Agilent Technologies 5800) was used to quantify seven elements in their soluble form: sodium (Na), calcium (Ca), potassium (K), magnesium (Mg), manganese (Mn), iron (Fe), and sulphur (S). These elements were selected because they are water quality or pollutant markers.^17^ Water samples were acidified with HNO_3_, to a final concentration of 2% (v/v), prior to analysis. Further information regarding the ICP-OES analysis can be found in the ESI‡ (Section S2).

### 2.3 Dissolved organic carbon (DOC)

Dissolved organic carbon (DOC) concentration (non-purgeable organic carbon) in filtered samples was assessed by a Total Organic Carbon L-series (TOC-L_CPH_) analyzer with an ASI-L autosampler (Shimadzu, Japan).

### 2.4 Fluorescence excitation–emission spectroscopy (FEEM)

Fluorescence excitation–emission spectroscopy was performed to characterise the differences between each sample's organic compound composition. The spectrofluorometer used in this study was a Horiba Duetta-Bio, equipped with a quartz cuvette (3.5 mL). The instrument was configured as described in the ESI‡ (Section S2). Data was collected and exported using the EZSpec software package. Data post-treatment and peak picking was performed using R (ver. 4.1.3) and the package staRdom (ver. 1.1.25).^28^ Due to the relatively small number of samples, we decided to limit our FEEM data discussion to peak picking. The literature was used to select known excitation–emission (EEM) peak locations of interest,^29^ such as peak C, (ex340/em440) an indication of the amount of humic-like (recalcitrant) compounds, and peak BIX (em380 nm divided by em430 nm at ex310), an indicator of autotrophic productivity.^30^ Further FEEM peak discussion can be found in Fig. S2 and S3.‡

### 2.5 Sensor fabrication

Metasurfaces were fabricated using a standard top-down electron-beam lithography process. Commercial microfluidic chambers (Microfluidic Chip Shop) were attached to the device, and the nanopatterned surfaces were chemically modified in the microfluidic chambers with self-assembled monolayers of functional thiol molecules (R–SH, Table S2, Fig. S3 and S5‡). Functionalisation took place using 10 mM concentrations for 1 hour, followed by flushing the microfluidic channel with 1 mL of pure solvent (EtOH or water). After modification, each channel was connected in series to form a single channel (Fig. S4b‡). The total system volume when all connected was measured to be ∼270 μL. More information on the fabrication process can be found in the ESI.‡

### 2.6 Sensor measurement

A custom-built microscope with a programmable *XY*-translational stage (ThorLabs) was used to measure transmission spectra across each nano-tastebud (Fig. S8‡). Light from a broadband LED (10 dB, 470–850 nm range, MBB1F1, ThorLabs) was used to probe each nano-tastebud. The transmitted light was collected by a 10× objective and coupled to a StellarNet Microspectrophotometer (StellarNet Blue Wave). Each nano-tastebud was measured five times in a cruciform pattern from a central measurement point (Fig. S9‡). A syringe pump was used to flow samples through the microfluidic channels. Starting with DI water, light/dark references and sensor readings were acquired under a flow rate of 50 μL min^−1^. Between samples, the microfluidic channels were flushed with 500 μL of DI water followed by 400 μL of sample at 200 μL min^−1^. Transmission minima were calculated using a second derivative high order polynomial fit of the data, and the shift was calculated based on the change in resonance from DI water.

### 2.7 Statistical analysis

JMP17 software was used for all statistical analysis. Two matrices of data were analyzed *via* principal component analysis (PCA). For both matrices, the rows corresponded to the samples tested. The columns in the analytical chemistry matrix (Table S3‡) corresponded to ICP-OES, DOC, and FEEM (peak C and BIX) results. The columns in the sensor matrix (Table S4‡) corresponded to the transmission resonance shift from DI water for each NTB (Fig. 3a). Linear discriminant analysis (LDA) with *k*-fold cross validation (*k* = 5) was used on the sensor dataset to estimate accuracy and generate the ROC curves (ESI‡ Section S9).

## 3. Results and discussion

### 3.1 Chemometric water sample analysis

The 25 water samples (10 INF, 12 EFF, 3 TAP) were aliquoted and only thawed as needed for analysis. To better understand the parameter space of the water samples under analysis, we undertook inductively coupled plasma optical emission spectroscopy (ICP-OES) to identify the levels of the most common types of elements monitored for DWTW,^1^ and used an organic carbon analyzer to quantify the dissolved organic carbon (DOC).

**Fig. 2:**
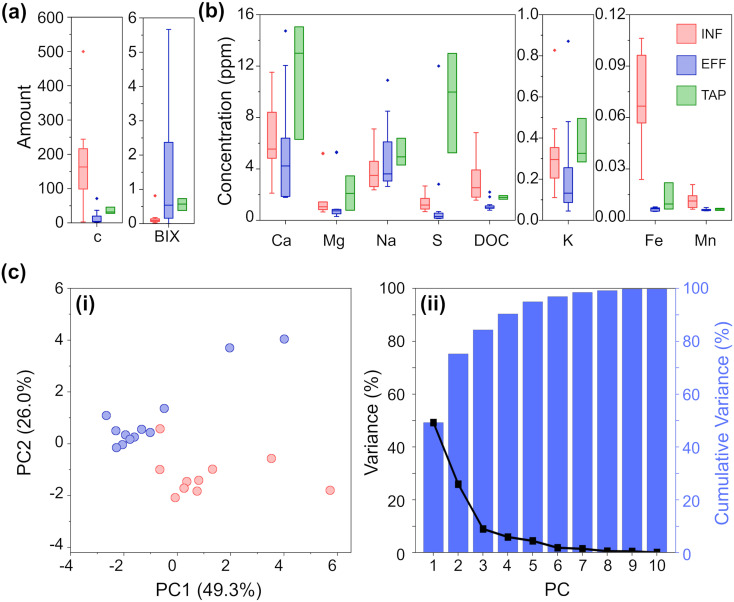
(a) FEEM analysis grouped by water sample type for peak C and bix. (b) ICP-OES and DOC analyses grouped by water sample type (concentrations are in ppm). (c) PCA of the analytical chemistry results for INF and EFF: (i) scatterplot of the first and second principal components (PC) and (ii) a plot of the variance and cumulative variance as explained by each PC.

Fig. 2 summarizes the results of the analytical chemistry measurements (a full breakdown by individual sample is provided in Fig. S1 and S2‡). DOC is an important parameter to assess, especially in the effluent and tap water, as a high DOC concentration could lead to microbial growth in water pipes and unwanted interaction with chlorinated compounds which can lead to the formation of disinfection by-products (DBPs). These DBPs, such as trihalomethanes, haloacetic acids, and chlorite, can pose health risks when consumed. Given the origin of our samples (all collected from surface water treatment works) and the significance of DOC in the sample set, we employed fluorescence excitation–emission spectroscopy (FEEM) to differentiate the water samples based on the composition of their dissolved organic matter (DOM). By utilizing FEEM in combination with chemometric techniques like peak picking, we gained insights into the dynamics of DOM. Influent surface water contains humic acid-like compounds (peak C) (Fig. 2a), which is not surprising considering the nature of the Scottish peaty soil. In Scotland, high concentration of DOC and humic acid-like compounds mostly occurs in upland freshwater in the northern and western parts.^31^ A higher peak BIX in effluent samples (Fig. 2a) highlighted an autotrophic carbon production during the treatment process, without increasing the overall DOC concentration significantly.

As expected, the DWTW process reduces the concentration of analytes to drinking water standards.^17^ For almost all the analyzed ions, the TAP samples exhibit greater variation compared to both INF and EFF samples. Interestingly, the variation in TAP samples is more similar to INF samples than EFF samples. This observation is likely due to additives that buffer the pH of the treated water, and to the water being exposed to multiple uncontrollable factors as it moves from the EFF stage through the distribution systems to reach households. The most notable differences between INF and EFF are DOC, Fe, S, Mg, and Mn ion concentrations, where INF on average has significantly higher TOC, Fe ion, and S ion concentrations and slightly higher levels of Mg and Mn ion concentrations than EFF. While the concentration of all analytes is generally higher for TAP than EFF, both water types have significantly reduced concentrations of Fe and Mn compared to INF.

Based on these quantitative data, we could clearly observe the difference between INF and EFF samples. To further exemplify this, we also performed PCA. PCA was chosen as an initial method of analysis for its ability to model the data by maximizing between sample variance, without bias (independent from the knowledge as to any identifying characteristics *i.e.* ‘type’ INF/EFF of each sample).^24,32,33^ The resulting scatterplot (Fig. 2c) of the first two principal components (PCs) for the chemical analysis shows INF (red) and EFF (blue) and explains 75.3% of the total variance. From the PCA, we can clearly see clustering amongst the INF samples and clustering amongst the EFF samples. From the Eigenvectors corresponding to the first three PCs (Fig. S12a, Table S5a‡), we see that PC1 is most influenced by K, Ca, Mg, DOC, and peak C; PC2 is most influenced by Fe and Na; and PC3 is most influenced by FEEM-BIX and S. We attribute these variations to the raw water sample location and variations in the methods of drinking water treatment. Even with the high chemical composition variability from location to location, the PCA shows two separate clusters corresponding to raw surface water and effluent water samples.

### 3.2 Design of a plasmonic nano-tastebud array for testing water

Based on this knowledge we constructed eight nano-tastebud regions, each comprising an array of gold nanostructures modified with a different cross-reactive chemical monolayer (Fig. 1, see Table S2 and Fig. S5‡ for further information on the chemical modifications). A short thiolated nitrilotriacetic acid derivative (NTA) and glutathione (GLU) were chosen for their zwitterionic properties and abilities to form complexes with particularly Fe and Mn, but also Ca, Mg, and Na ions *via* chelation.^34–36^ Dodecanethiol (DDT) was chosen for its hydrophobic nature and perfluorodecanethiol (PDFT) was chosen for its perfluoric nature (these nano-tastebuds are most likely to interact only with other hydrophobic or perfluoric compounds, respectively).^37,38^*Para*-modified derivatives of thiophenol, aminothiophenol (ATP), mercaptobenzoic acid (MBA), mercaptophenolboronic acid (MPBA), and nitothiophenol (NTP) were chosen because their positive and negative charges at pH 7 can (1) promote interaction with ions and molecules of the opposite charge, and (2) withdraw/donate electrons to the phenol rings, themselves, which then promotes pi–pi stacking of the phenol groups with suitable electron rich or electron poor pi-systems in organic carbon. The shift in resonance before and after thiolation is shown in Fig. S7.‡

**Fig. 3:**
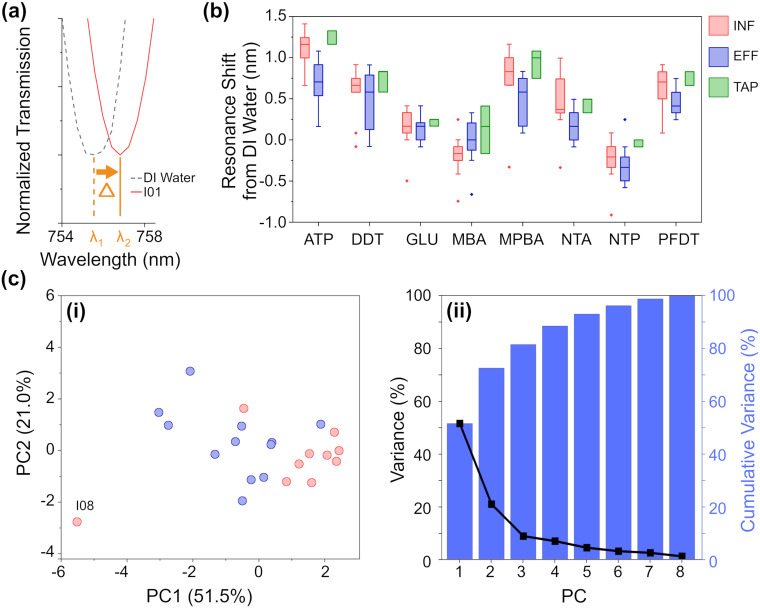
(a) Example of a resonance redshift from DI water (ATP chemistry, sample I01). (b) Resonance shift from DI water of each NTB grouped by water sample type. (c) PCA of the resonance shift (average shift from DI water) for INF and EFF: (i) scatterplot of the first and second principal components and (ii) a plot of the variance and cumulative variance as explained by each PC.

A calibration baseline of DI water was measured, followed by each sample, with the resonance shift from DI water determined based on five replicate measurements (Fig. 3a, full data shown in Fig. S10‡). Samples were filtered prior to analysis to prevent biofouling and clogging of the microfluidic platform. As can be seen in the summarized results (Fig. 3b), each NTB has its own, unique response to the samples. Overall, there is quite a lot of variation between and within each ‘type’ of water, which is not surprising given the large variation also seen in the analytical chemistry results. Except for MBA and NTP, there is an overall redshift (the transmission minima shift to higher wavelengths; example shown in Fig. 3a) for all samples. For ATP, MPBA, and PFDT, the redshift has a larger magnitude from EFF to INF to TAP samples, respectively. For ion-binding NTA, the magnitude of red shift is highest for INF, followed by TAP, then EFF, which correlates with the increasing concentration of Fe and Mn ions seen in the ICP-OES results. For MBA, INF samples cause a blue-shift, whereas EFF and TAP samples caused a small red-shift. For NTP, most samples generally cause a blue-shift, with a higher magnitude of blue-shift for EFF than INF, while TAP is almost unchanged. ATP, MPBA, NTA, and PFDT have the most variation between INF, EFF, and TAP, while GLU and DDT have the least. From the full breakdown NTB response to each individual sample (Fig. S10‡), INF sample I08 appears to stand apart from the others. Altogether, these variations in response demonstrate the partially-selective properties of the sensor. The PCA scatterplot of the first and second principal components of the resonance shifts (Fig. 3c, explaining 72.5% of the total variance) shows INF (red) and EFF (blue) both cluster as hoped, and again INF sample I08 (labelled, Fig. 3c) is somewhat of an outlier.

From the eigenvectors corresponding to the first three PCs (Table S5b, Fig. S12b‡), we can ascertain that the split between INF and EFF in the PCA is driven evenly by all the sensing elements, with ATP, DDT, and MPBA having a slightly stronger effect. From the eigenvectors corresponding to the PCA for the chemometric data, this split is driven by Fe, K, DOC, BIX, and peak C so there may be some correlation between these chemometric measurements and the aforementioned NTB elements.

### 3.3 Robust water classification with a plasmonic array

While PCA shows that clustering is possible, to enable practical analysis of unknown water samples and determine if they are more like influent or effluent water, a supervised analysis technique is required.^24,33^ LDA was chosen for its ability to maximize separation between known clusters while minimizing the variance within each cluster.^33^

**Fig. 4:**
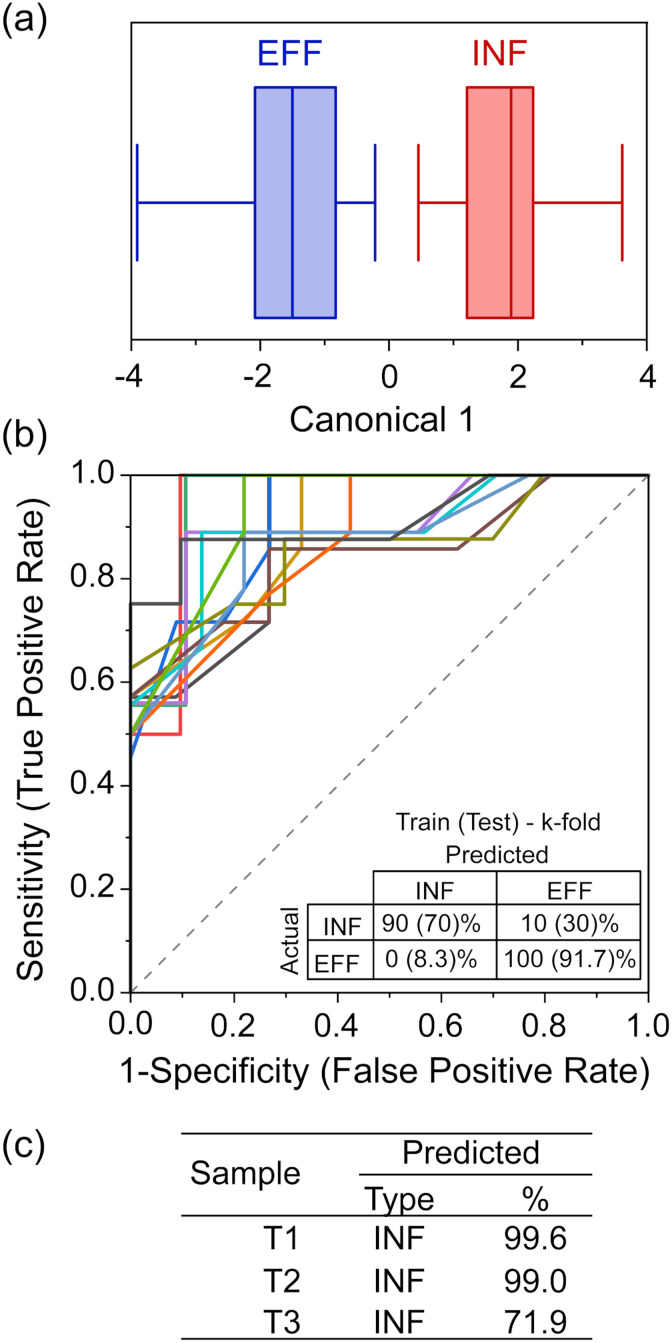
(a) LDA of the sensor response (input is average resonance shift from DI water per NTB per sample, with *k*-fold cross validation, *k* = 5) comparing INF and EFF groups, only. (b) ROC curves and (inset) the averaged (25 iterations) classification table for the accuracy of the training set and the validation set. (c) The predicted classification and percent probability for the three excluded tap water samples, using the LDA model.

From the LDA (Fig. 4a) and the corresponding classification matrix (Table S4a‡), the sensor appears to have 100% accuracy in differentiating between INF and EFF. We further tested the robustness by validating its classification capabilities. To do this, we used *k*-fold cross-validation (*k* = 5) and iterated the selection of the training set and validation set, 25 times, producing a series of receiver operating characteristic (ROC) curves and classification matrices. The average area under the ROC curves (where 1 is a perfect classification, and 0.5 is no better than chance) (Fig. 4b) is 0.897 (with the worst-performant model being 0.8442), indicating robust discriminatory capability. From the averaged classification matrix of the training set (Fig. 4b, inset), the sensor has an overall accuracy of 95.5% (90% successful classification of INF and 100% of EFF). From the averaged classification matrix of the validation set (the held back 1/5 of the data), the sensor has an overall accuracy of 81.8% (70% successful classification of INF and 91.7% of EFF).

Compared to EFF samples, TAP samples are sampled after having travelled through the distribution systems for varied distances; additionally, chlorination and addition of buffering agent are likely to have happened as the water exited the DWTW, changing the nature of the organic matter. During travel in the pipe bacterial growth may occur, with biofilm detachment and secondary contamination also affecting the chemical composition of the water. The chemical characterisation of the three TAP samples (Fig. 2) showed that they had different and more varied signatures than the EFF and INF samples. They had low DOC but high metal concentrations and an increased BIX compared to the EFF samples, to which they are expected to be more similar. This could indicate a greater microbial content or activity in the TAP samples. We therefore chose to analyse the tap water samples and test whether changes in the chemical composition of treated water could be detected by the device. When LDA was used to classify these samples using the nano-tastebud data, T01, T02, T03 were all predicted to be “INF” type with 99.6%, 99.0%, and 71.9% probability, respectively (Fig. 4c). This last value of 71.9% can be explained by the difference in concentration of the measured compounds. Fig. S1 and S2‡ show that T03 contains a higher iron concentration, lower BIX, and higher peak C (humic-like compounds) compared to the two other TAP samples. The determinant analysis could not be trained for TAP as a category due to the low number of samples; nonetheless, the sensor could differentiate effluent samples just after treatment, and tap water samples that had changed in composition on the way to the sampling site. This demonstrated that the nano-tastebuds have the potential to be used as a sensor to signal changes in water composition that could prompt an intervention. The misclassification of some tap samples as influent water could in future be solved by expanding the range of sensor elements and modifications used, particularly if one or more of these were tailored to respond to chlorinated compounds (in any real-world scenario it would be most beneficial to have sensor combinations dedicated to specific tasks/sites; different sensor designs used for detecting failures in treatment sites and for measuring composition of tap water, for example).

Although real-world water monitoring would always take place in an inherently dynamic environment, we believe that, as a proof-of-concept, the technology shows promise for real-world use. The power of the sensors lies in their ability to measure change from a baseline; change that could indicate a treatment system failure, or, if the sensor was tailored to consumer deployment, could alert to compositional changes developed in the journey to the tap. As a result, we believe that variants of this sensor system could be used throughout the treatment and distribution system to detect issues such as bacterial regrowth, biofilm detachment, or presence of organic pollutants (including chlorinated by products). Further work will involve understanding the sensitivity of the technology, if measured water changes can be correlated with issues known to influence water composition (*e.g.* AOC [assimilable organic carbon] content), how early specific changes can be detected, and ultimately, whether treatment malfunctions can be identified before water composition becomes hazardous to health.

## Conclusion

In this manuscript, we successfully demonstrate an optical nano-tastebud sensor that can discriminate between treated, un-treated, and tap water samples from various sites across Scotland. While further testing and development of the nano-tastebud array is warranted, once fully developed we believe that such a system could provide an early warning system for impending system failures, going beyond the standard ‘sample and test’ monitoring methods broadly used worldwide.

## Author contributions

Conceptualization: JRS, BP, LS, WTS, CGL, WJP, and AWC; data curation: JRS, BP, LS, BLA, CC, and WJP; formal analysis: JRS, BP, and WJP; funding acquisition: JR, WTS, CGL, WJP, and AWC; investigation: JRS, BP, LS, BLA, IC, and KM; methodology: JRS, BP, LS, and KM; project administration: WTS, CGL, WJP, and AWC; resources: ASK, WTS, CGL, WJP, and AWC; software: JRS and CC; supervision: ASK, JR, WTS, CGL, WJP, and AWC; validation: JRS, BP, LS, IC, CGL, WJP, and AWC; visualization: JRS, WJP, and AWC; writing – original draft: JRS, BP, WJP, and AWC; writing – review & editing: all authors.

## Conflicts of interest

Authors J. R. Sperling, I. Christie, W. J. Peveler, and A. W. Clark are named on patent applications that involve a similar sensing concept. International (WO) Patent Application Numbers PCT/EP2021/052628 and PCT/EP2023/063146.

## Supplementary Material

EN-010-D3EN00565H-s001
