# Supplementary material for: A cross-reactive plasmonic sensing array for drinking water assessment
Source: Environ Sci Nano. 2023 Nov 13;10(12):3500–8. doi: 10.1039/d3en00565h (PMC10702629; doi:10.1039/d3en00565h)
Supplement: EN-010-D3EN00565H-s001 [file EN-010-D3EN00565H-s001.pdf]

Electronic Supplementary Material (ESI) for Environmental Science: Nano.
This journal is © The Royal Society of Chemistry 2023

A cross-reactive plasmonic sensing array for drinking water assessment. Supporting Information (SI)

Justin R. Sperling *[,a], Baptiste Poursat *[,a], Laurie Savage [a], Iain Christie [a], Calum Cuthill [a], Badri Aekbote [a], Katie McGuire [b], Affar S. Karimullah [b], Jill Robbie [c], William T. Sloan [a], Caroline Gauchotte-Lindsay [a], William J. Peveler [b], Alasdair W. Clark [a]

[a] James Watt School of Engineering, University of Glasgow, Glasgow, United Kingdom
[b] School of Chemistry, University of Glasgow, Glasgow, United Kingdom
[c] School of Law, University of Glasgow, Glasgow, United Kingdom

Contents

1. Sample collection 2
2. Analytical chemistry 2
3. Synthesis of custom nitrilotriacetic acid thiol 4
4. Sensor fabrication 6
5. Sensor thiolation 7
6. Transmission microscopy 10
7. Nano-tastebud sensing of Scottish Water samples 11
8. Principal component analysis (PCA) 12
9. Linear discriminant analysis (LDA) 13
10 References 14

1. Sample collection

Scottish Water provided 10 samples of influent (INF, I01-I10), 12 samples of effluent (EFF, E01-E12), and 3 sample of tap (TAP, T01-T03) water from various drinking water treatment works sites and randomly selected consumer taps across Scotland. The exact location of each sample collected is not provided due to an NDA with Scottish Water. Samples were frozen upon collection. Prior to analytical chemistry analysis and transmission microscopy, samples were defrosted overnight at 4°C and then filtered using a 0.22 µm filter.

2. Analytical chemistry

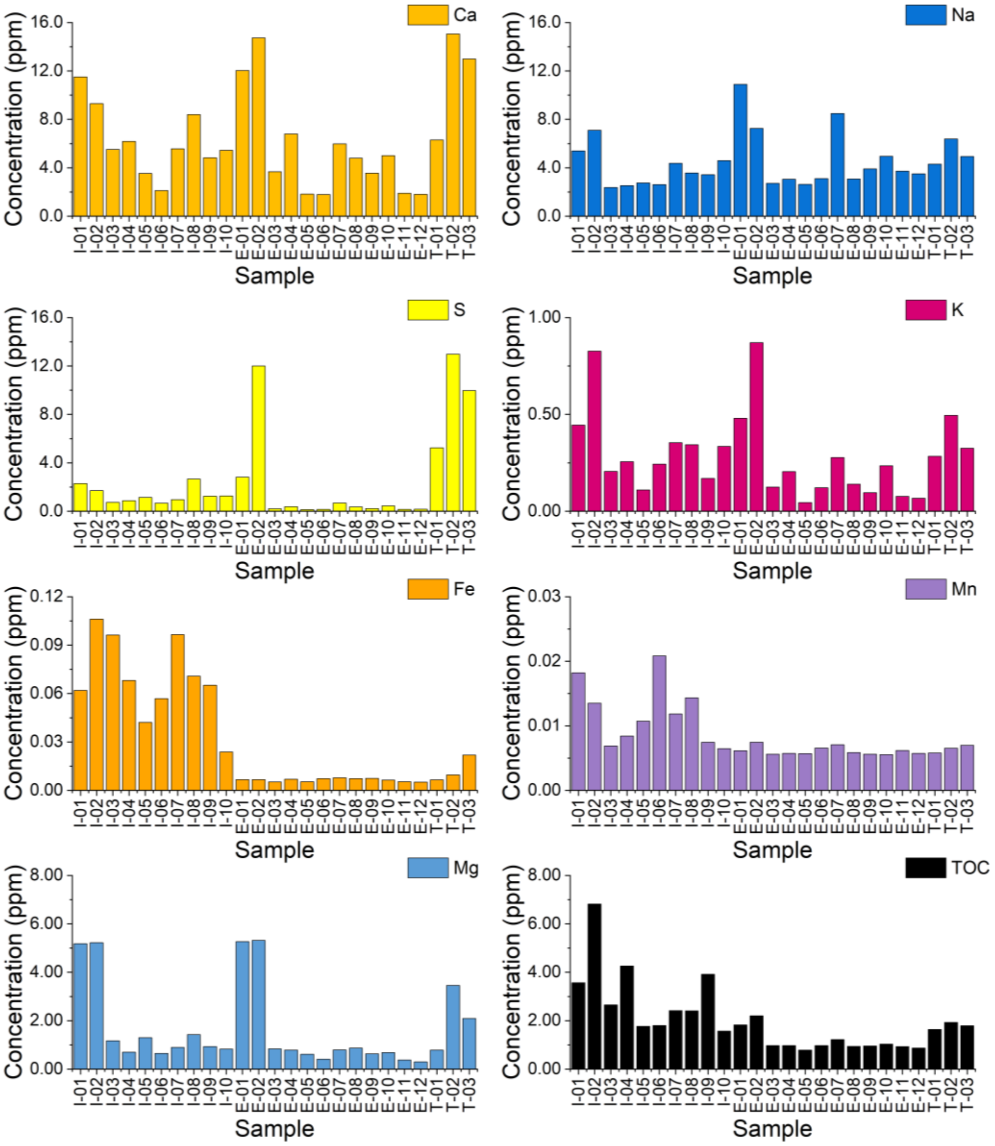

Figure S1 ICP-OES and DOC analysis results of all 25 samples collected from Scottish Water. From top left to bottom left, concentration in ppm for Ca, Na, S, K, Fe, Mn, and Mg (ICP-OES). In bottom right, DOC in ppm.

Inductively coupled plasma optical emission spectroscopy (ICP-OES, Agilent Technologies 5800) was used to assess the levels of the following ions in the filtered samples: Na, Mg, Mn, K, Fe, S, and Ca. Water samples (10 mL) were acidified to 2% with HNO_3. Four calibration standards containing a known concentration of analytes were prepared with the 2% HNO_3 acidified, ultra-pure water. Calibration standards concentration ranged from 0.05

to 10 ppm (0.05, 0.1, 1, 10). A blank containing no analytes was prepared under the same condition. The ICP-OES connected to Agilent SPS 4 autosampler was used to quantify the standards and the samples. Each sample was injected 6 times for 17 sec and read for 10 sec. A radial viewing mode was used. Analyzed element wavelength for Na, Ca, K, Mg, Mn, Fe, S can be found in **SI table 1**. Weighted linear regression was used to achieve calibration fit.

Table S1. Wavelength (nm) of analyzed element.

Element	Wavelength (nm)
Fe	238.204
Mn	257.610
S	181.972
Ca	396.847
K	766.491
Mg	279.553
Na	589.592

Dissolved organic carbon (DOC) concentration (non-purgeable organic carbon) in filtered samples was assessed by TOC-LCPH analyzer with an ASI-L autosampler (Shimadzu, Japan). Control of 2.5 ppm and ultra-pure water was used to assess the quality of the measurement.A full breakdown of the concentrations for each sample is shown in **Figure S1**. Comparing the INF to EFF

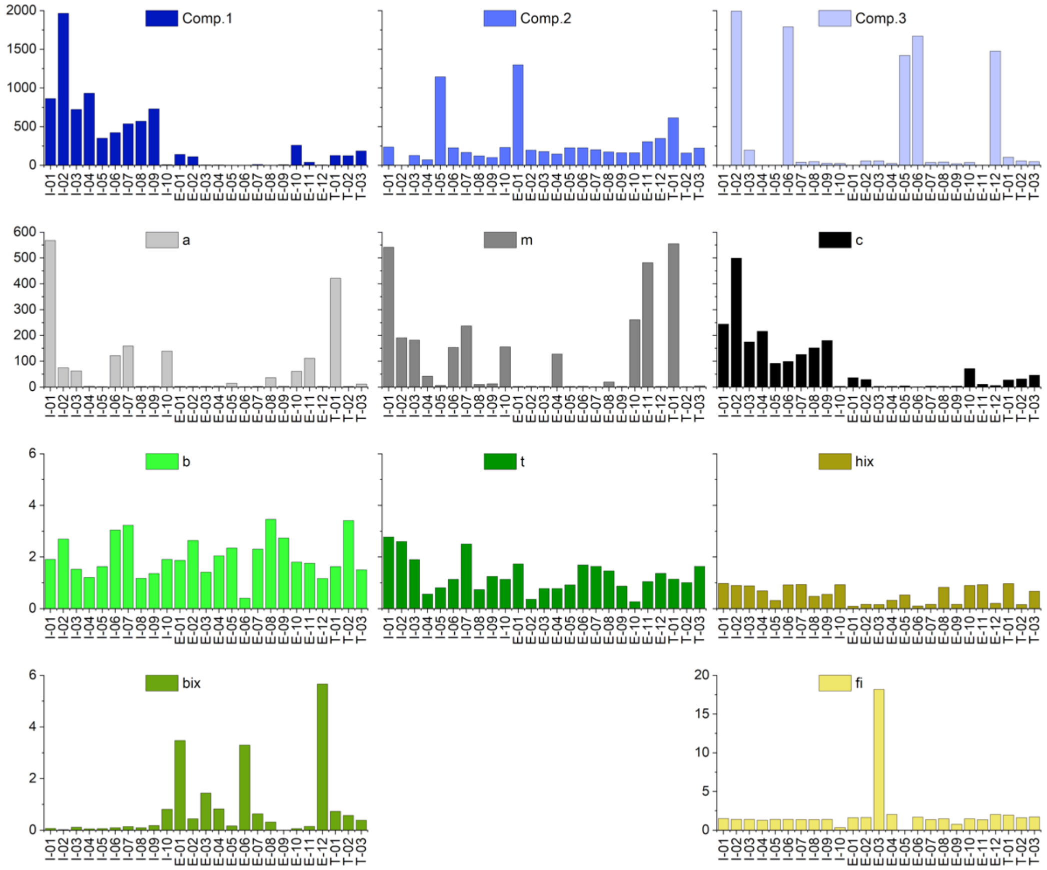

Figure S2 FEEM analysis of results of all 25 samples collected from Scottish Water.

Fluorescence excitation-emission spectroscopy (FEEM) of each sample spectroscopy was performed on a Horiba Duetta Bio and data exported form the EZSpec software package. instrument was configured with settings as described here: The excitation range was set from 250 to 470 nm with 10 nm increments, while the emission range was set from 280 to 550 nm. To account for the Inner Filter Effect (IFE), an automatic correction was applied. The excitation and emission band pass were both set at 5 nm, and the integration time for measurements was set to 1 second with a single detector accumulation. During the measurement, each sample was prepared by loading 3 mL into the cuvette, which was then inserted into the spectrofluorometer. Absorbance and fluorescence measurements were taken sequentially, with automatic correction for IFE. Prior to each sample measurement, a blank (pure water) was measured and its signal was subtracted from the sample signal. Additionally, the samples were Raman calibrated using an excitation wavelength of 350 nm, following the previously described method.

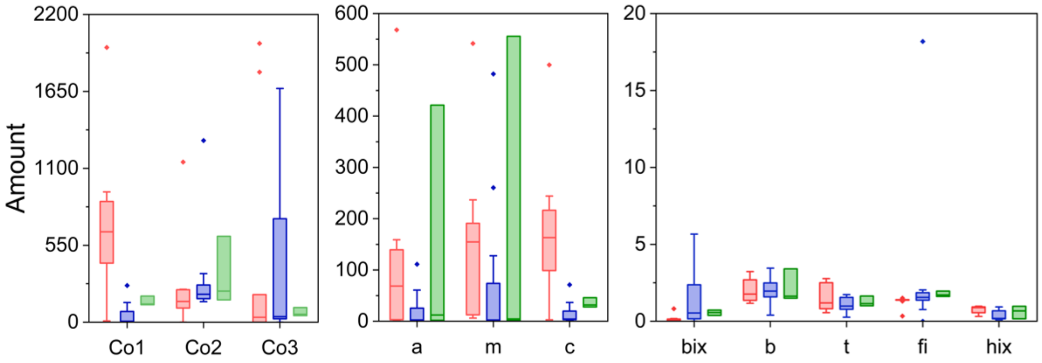

Figure S3 Summary figure of FEEM results grouped by INF, EFF, and TAP.

3. Synthesis of custom nitrilotriacetic acid thiol

Reagents were used as supplied from Sigma Aldrich and Fisher Scientific without further purification. Water used was deionised (>15 MΩ) and non-aqueous solvents used were of analytical grade. Thin layer chromatography was performed on Merck aluminium backed silica gel 60 F254 plates. Visualisation was achieved with UV light, iodine on silica, or $KMnO_4$ in basic aqueous solution.

^1H NMR and ^{13}C NMR were collected on Bunker AVI 400 spectrometers in deuterated solvents as noted. Chemical shifts are recorded in ppm, relative to the residual protonated solvent. Coupling constants are recorded in Hz. Mass spectra data for each compound were recorded on a Bruker microTOFq system using positive mode electrospray ionisation (ESI). Molecular ions or other major ion peaks are reported as *m/z*.

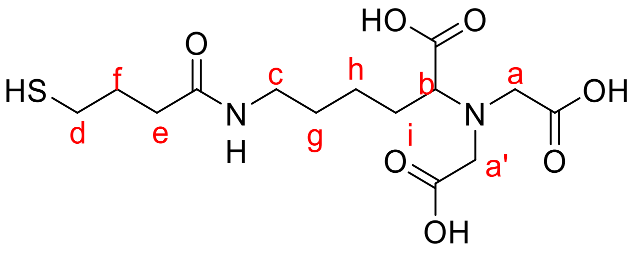

Figure S4. Thiol-Nα,Nα-Bis(carboxymethyl)-L-lysine (HS-NTA)

HS-NTA was synthesised in a method adapted from Du Roure *et al.*[1] Nα,Nα-Bis (carboxymethyl)-L-lysine hydrate (0.1 g, 0.38 mmol) , $NaHCO_3$ (0.1 g, 1.19 mmol) and 4-butryothiolactone (51 µL, 0.59 mmol) were dissolved in 5 mL water and heated to 72 °C for 3 days. The resultant solution was cooled to RT and acidified to pH 3 with 100 µL of glacial acetic acid. The solvent was removed under reduced pressure. The resultant oil was crystalized in EtOH and the crystals were washed with cold 10 mL EtOH and 10 mL hexane. This yielded the product as a white powder (50 mg, 34.72 %). Observed analytical data were in accordance with literature values.

^1H NMR (400 MHz, $CDCl_3$) δ 3.70 (m, 5H, H_a, $H_{a'}$, H_b), 3.14(br, 2H ,H_c), 2.67 (t, J=6.84 Hz, 1H, H_d), 2.48 (t, J=6.84 Hz, 1H, H_d), 2.29 (t, J=7.90 Hz, 2H, H_e), 1.72-1.98 (m, 4H, H_f, H_g, H_h, H_i), 1.35-1.64 (m, 4H, H_f, H_g, H_h, H_i).

^{13}C NMR (133 MHz, D_2O) δ 175.69, 68.36, 61.70, 57.41, 38.90, 36.99, 34.30, 30.21, 28.08, 26.67, 24.69, 23.72, 13.22.

MS (ESI+) *m/z* 365.14 ($[M+H]^+$ $C_{14}H_{24}N_2O_7SH^+$ calc. 365.41).

4. Sensor fabrication

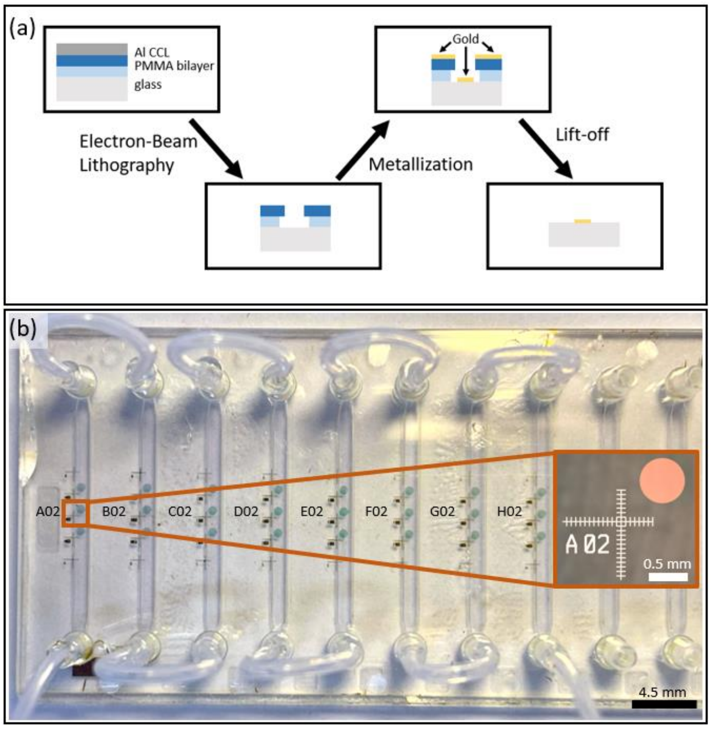

Figure S5 (a) Electron-beam lithography fabrication flow diagram. **(b)** Photo of the fully assembled sensor. (inset) Brightfield microscopy (5x) of one element in the sensing array with alignment reticule and label to the left and circular extent of nanostructure array to the right.

Devices were fabricated using a standard top-down electron-beam lithography process. As shown in **Figure S5a,** a poly(methyl methacrylate) (PMMA) bilayer (Layer 1: AR-P 642.04 (200k, 4%), anisole, 100 nm thick; Layer 2: AR-P 679.02 (950k, 2%), ethyl lactate, 70 nm thick) was spun on borosilicate glass (PI-KEM Ltd.) followed by the evaporation of a 20 nm aluminum (Plassys MEB 550S) charge conduction layer (CCL). A Raith EBPG 5200 electron-beam lithography tool was used to pattern multiple 500 um diameter circular extents of nanostructure (130 nm x 130 nm squares with 390 nm periodicity in XY) and reticule alignment markers in an array with periodicity of 1.5 mm in Y and 4.5 mm in X. Post-patterning, the CCL was removed using Microposit MF CD-26 (Shipley). Samples were then developed in 2.5:1 isopropyl alcohol (Scientific Laboratory Supplies, Ltd.) to methyl isobutyl ketone (Merck Chemicals) followed by evaporation of 2 nm titanium adhesion layer and 50 nm gold (Plassys MEB 550S) and PMMA/metal lift-off in acetone (Scientific Laboratory Supplies, Ltd.). Commercial microfluidic chambers (Microfluidic Chip Shop) were aligned then attached to the device. After thiolation, each channel was then interconnected to form a single channel. **Figure S5b** shows the fully assembled device.

5. Sensor thiolation

Table S2. List of surface chemistries used to modify the nanopatterned regions.

Modification	Abbr.	Solvent	Manufacturer
4-aminothiophenol	ATP	ethanol	Aldrich
1-dodecanethiol	DDT	ethanol	Sigma-Aldrich
L-glutathione	GLU	water	Alfa Aesar
4-mercaptobenzoic acid	MBA	ethanol	Aldrich
4-mercaptophenylboronic acid	MPBA	ethanol	Aldrich
Nitrilotriacetic acid thiol	NTA	water	custom fabrication (see SI Section 3)
4-nitrothiophenol	NTP	ethanol	Aldrich
1H,1H,2H,2H-perfluorodecanethiol / (heptadecafluoro-1,1,2,2-tetrahydrodecyl)triethoxysilane	PFDT	ethanol	Sigma-Aldrich / Gelest

The nanopatterned surfaces were chemically modified in the microfluidic chambers, with self-assembled monolayers of 10 mM concentrations of functional thiol molecules (R-SH, **Table S2**, **Figure S6**) for 1 hour, followed by flushing of 1 mL of pure solvent. A balance between thiolation reaction time and minimizing the exposure of the microfluidic channel adhesive degradation caused by ethanolic solution was necessary.

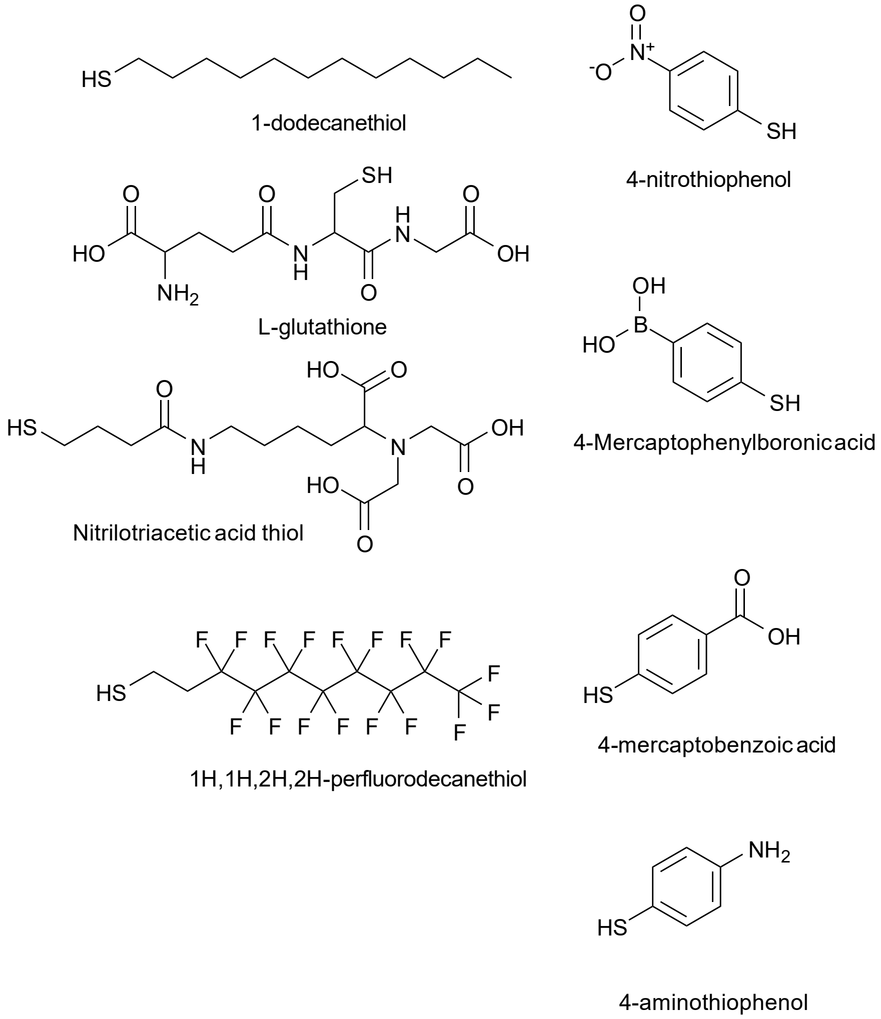

Figure S6. Chemical structure for the thiols (R-SH).

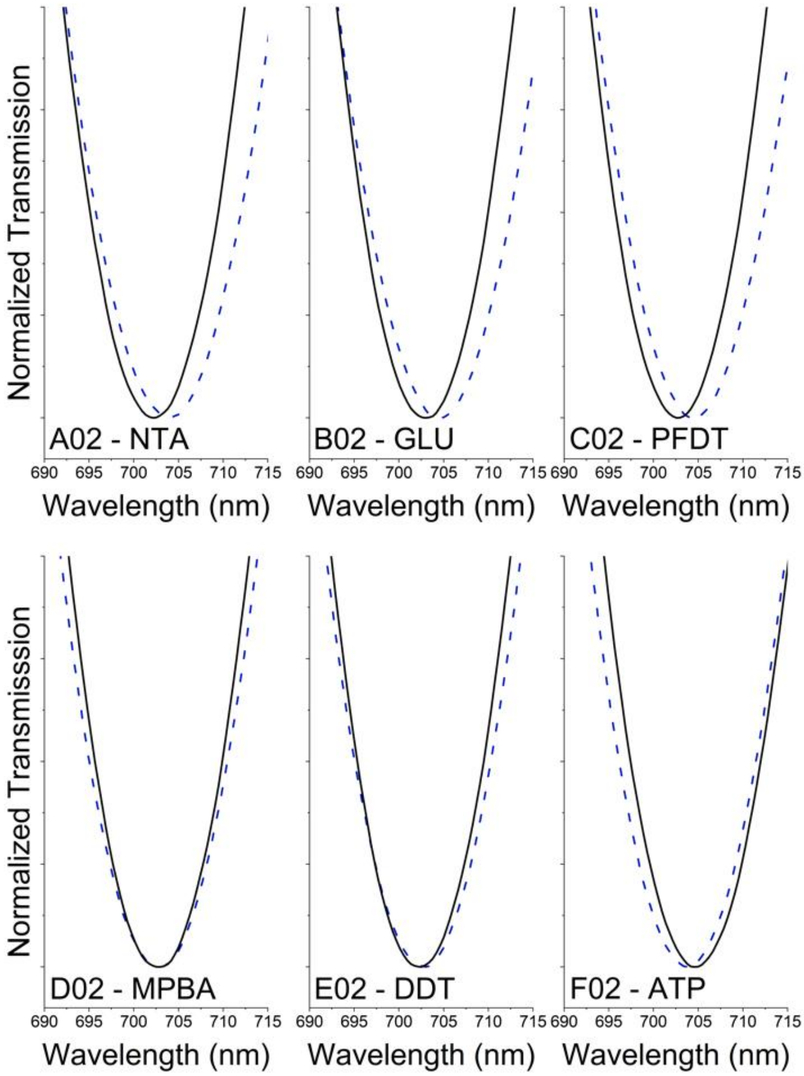

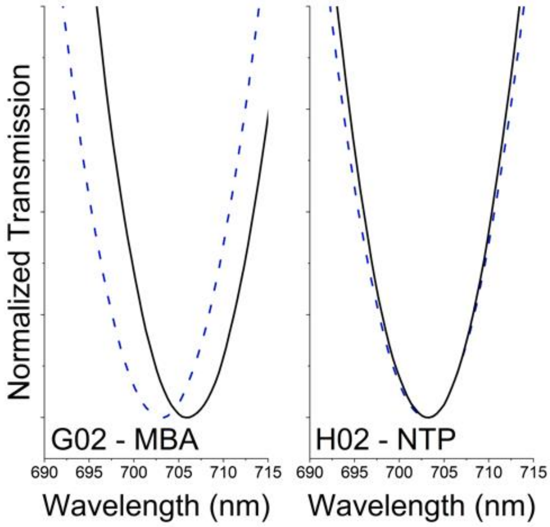

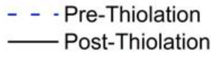

Chem	Pre-Thiol Peak (nm)	Post-Thiol Peak (nm)	Shift (nm)
NTA	704.0	701.7	−2.3
GLU	704.4	703.0	−1.5
PFDT	704.2	702.0	−2.2
MPBA	703.2	703.3	0.1
DDT	704.5	704.2	−0.3
ATP	703.0	703.7	0.7
MBA	703.3	706.0	2.6
NTP	704.0	704.2	0.2

Figure S7. Transmission resonance pre (dashed, blue) and post (solid, black) thiolation for each nano-tastebud. The table in the bottom right provided the identified resonance peak pre- and post-modification and the resulting resonance shift. To make the comparison easy, each spectra is smoothed (30 point moving average) and normalized.

The R-group of the thiols were chosen to provide a wide of a range of surface types, which promotes high cross-reactivity between nano-tastebuds. Exact selection was influenced by the results in the analytical chemistry analysis (**Section S2**). The transmission spectra shifts from the thiolation are shown in **Figure S7**. The resulting resonance shift is a unique combination of the number of thiols that attach and the refractive index, electron withdrawing/donating properties, and dipole moment of the thiol.[2–7]

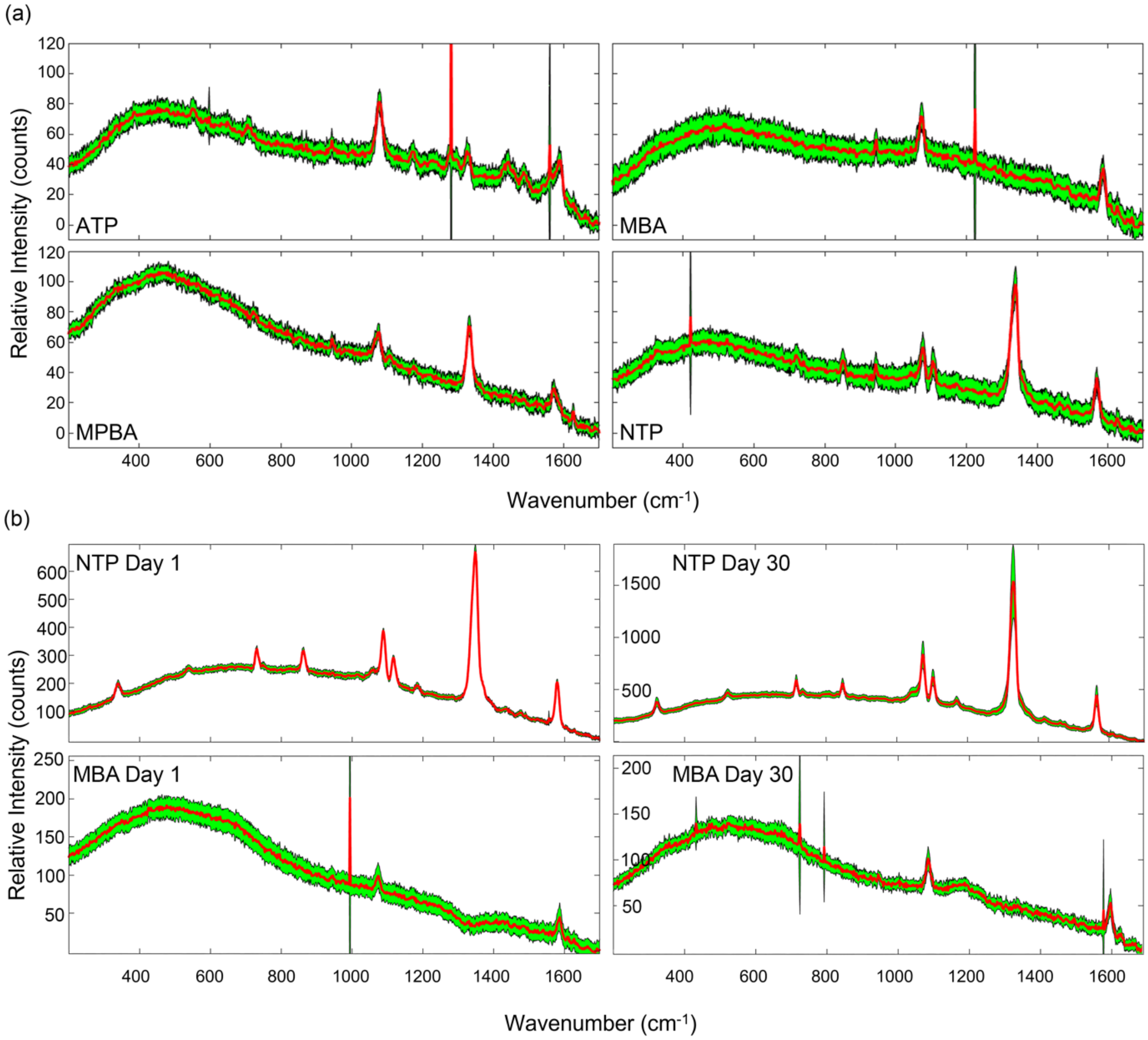

Figure S8. (a) Averaged Raman spectroscopy signal from 4x4 mapping of the four NTBs with phenol-containing thiols. Clock-wise from top-left: ATP, MBA, NTP, and MPBA. The red line is the average signal and the green outline represents the standard deviation from all 16 measurements. (b) Raman spectra showing the stability of the molecular modifications on sensors that have been stored in water for 30 days. Top row: A sensor identical to those

used in the main experiment, modified with NTP, showing no decrease in Raman signal over 30 days. Bottom row: A sensor identical to those used in the main experiment, modified with MBA, showing no decrease in Raman signal over 30 days.

In addition to looking at transmission spectra shift, we also looked at Raman spectroscopy signals once the NTBs were thiolated (**Figure S8(a)**). Raman spectra maps (4x4, 25 μm step in X and Y) were recorded using a NT-MDT NTEGRA Raman microscope with a 633 nm laser excitation (35 mW power) with 10 second accumulation time. Excitation and collection of Raman scattered light was done using a 20x objective with an estimated spot size of 100 μm. The averaged Raman spectra of the NTBs with the four phenol-containing thiols (ATP, MBA, MPBA, and NTP) are in good agreement with the literature[8–10]. The thiols present on the other four NTBs (DDT, NTA, GLU and PFDT) did not exhibit a Raman signal. We attribute this to the physical structure of the molecule itself combined with the low-magnification objective used in the setup. Also shown in **Figure S8(b)** are spectra from NTBs modified with NTP and MBA, before and after storage in water for 30 days. These results show that there is no measurable desorption of the monolayer over this period.

6. Transmission microscopy

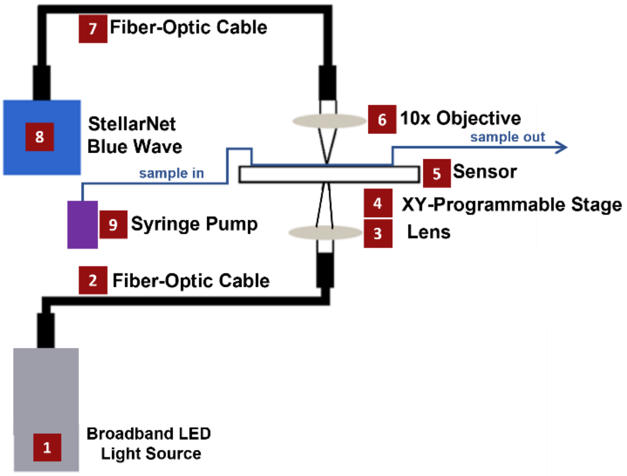

Figure S9. Custom-built micros*cope* (transmission-mode) with programmable XY-stage*, syringe pump microfluidics, and spectrophotometer*.

A custom-built microscope with a programmable XY-translational stage (ThorLabs) was used to measure transmission spectra across each nano-tastebud (**Figure S9**). Prior to measurements, reticules in the design were used to identify position and correct for angular rotation of the sensor. Light from a broadband LED (10dB, 470-850 nm range, MBB1F1, ThorLabs) was used to probe each nano-tastebud. The transmitted light was collected by a 10x objective and coupled to a StellarNet Microspectrophotometer (StellarNet Blue Wave). Each nano-tastebud was measured five times in a cross-like pattern (50 µm step, see **Figure S10**).

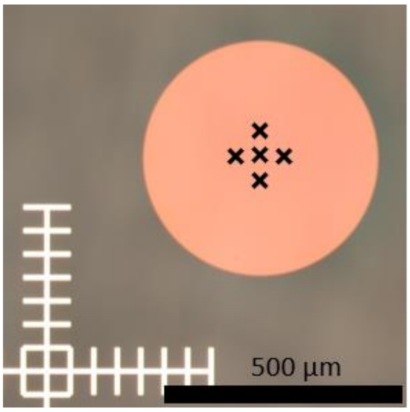

Figure S10. Cross-like pattern used for measuring five spots around a nano-tastebud. Each ‘x’ represents a 50 µm step.

Transmission resonance minima was calculated using the second derivative high order polynomial fit of the data and the shift was calculated based on the change in resonance from deionized (DI) Water.

To ensure easy visual comparisons for all figures showing transmission shifts, the plotted transmission spectra were smoothed (30-point moving-average) and normalized.

7. Nano-tastebud sensing of Scottish Water samples

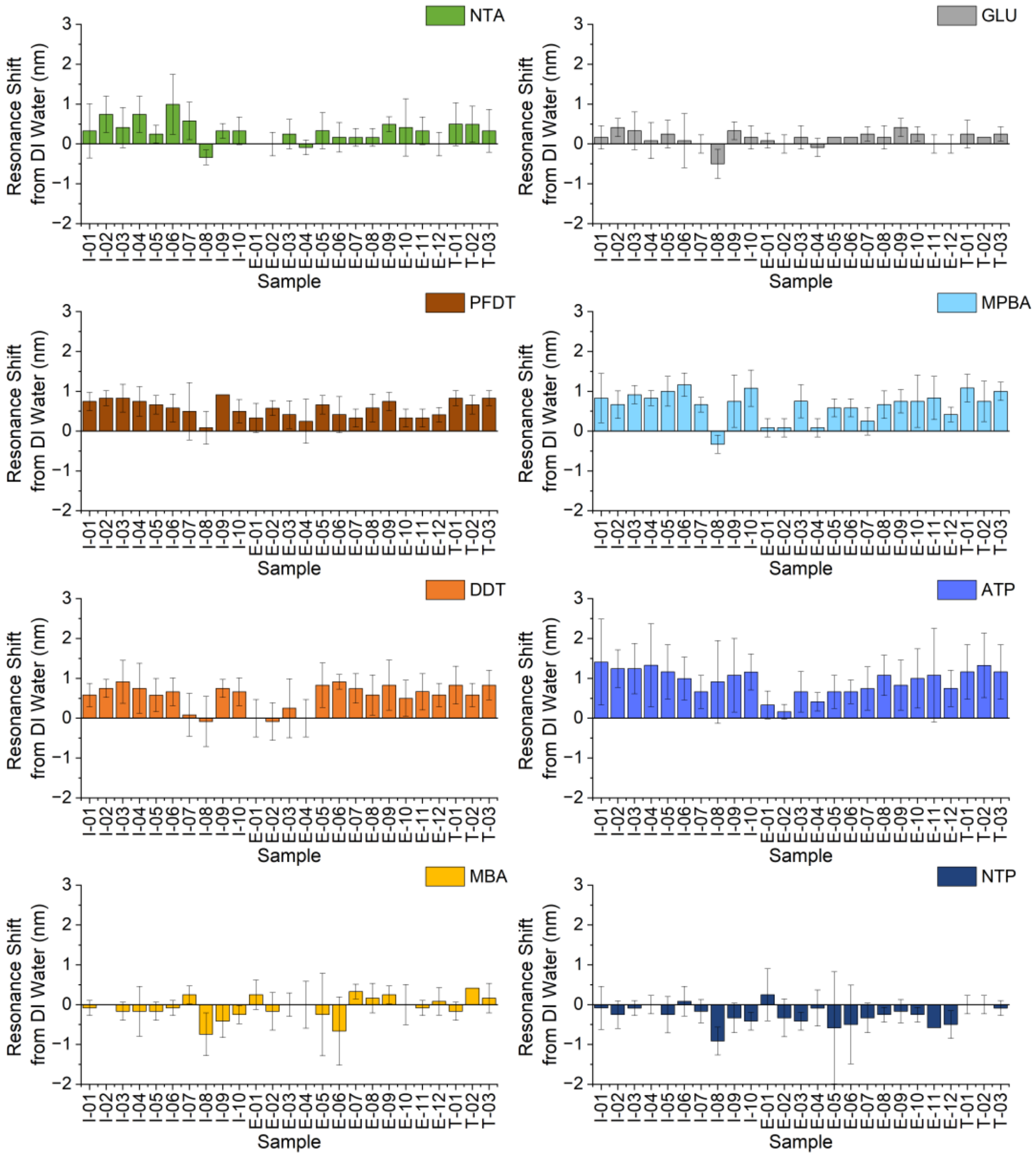

Figure S11. Average resonance shift from DI Water for each nano-tastebud when exposed to the 10, 12, and 3 samples of INF, EFF, and TAP water, respectively.

8. Principal component analysis (PCA)

Table S3. Data matrix used for PCA of the analytical chemistry results.

Sample	ICP-OES							DOC	FEEM	
**Sample**	**Ca**	**Fe**	**K**	**Mg**	**Mn**	**Na**	**S**	**DOC**	**peak C**	**BIX**
I01	11.52	0.06	0.45	5.18	0.02	5.40	2.30	3.57	244	0.07
. . .										
E12	12.04	7E-3	0.48	5.27	6E-3	10.9	2.81	1.83	6.04	5.66

Table S4. Data matrix for the PCA of the NTB response.

Sample	ATP	DDT	GLU	MBA	MPBA	NTA	NTP	PFDT
I01	1.41	0.58	0.17	-0.08	0.83	0.33	-0.08	0.75
. . .								
E12	0.33	2E-3	0.08	0.25	0.08	0.00	0.25	0.33

JMP17 software was used to run PCA of the analytical chemistry and NTB sensor. For both PCA, the rows corresponded to the sample tested. In the analytical chemistry PCA (**Table S3**), the columns corresponded to the ICP-OES, DOC, and FEEM (peak C and BIX) results. In the NTB sensor PCA (**Table S4**), the columns corresponded to the change in transmission resonance minima from DI water for each NTB (**Figure 3a**).

Table S5. PCA Eigenvector table for the first three principal components (PCs) of the (a) analytical chemistry and (b) NTB sensor.

(a)	PC1	PC2	PC3
Ca	0.38	0.27	-0.13
Fe	0.27	-0.45	0.10
K	0.42	0.17	-0.05
Mg	0.39	0.23	0.10
Mn	0.24	-0.31	-0.08
Na	0.25	0.39	0.26
S	0.28	0.34	-0.42
DOC	0.36	-0.28	0.23
FEEM-bix	-0.13	0.30	0.77
FEEM-c	0.34	-0.35	0.25

(b)	PC1	PC2	PC3
ATP	0.34	-0.36	0.44
DDT	0.39	-0.29	-0.37
GLU	0.40	0.14	-0.58
MBA	0.10	0.65	-0.16
MPBA	0.43	-0.10	0.25
NTA	0.41	0.10	0.38
NTP	0.21	0.58	0.34
PFDT	0.40	-0.03	-0.18

The corresponding Eigenvectors for the first three components of both PCAs are shown in **Table S5**. The Eigenvector plots for both the chemometric data and sensor data are shown in Figure S12.

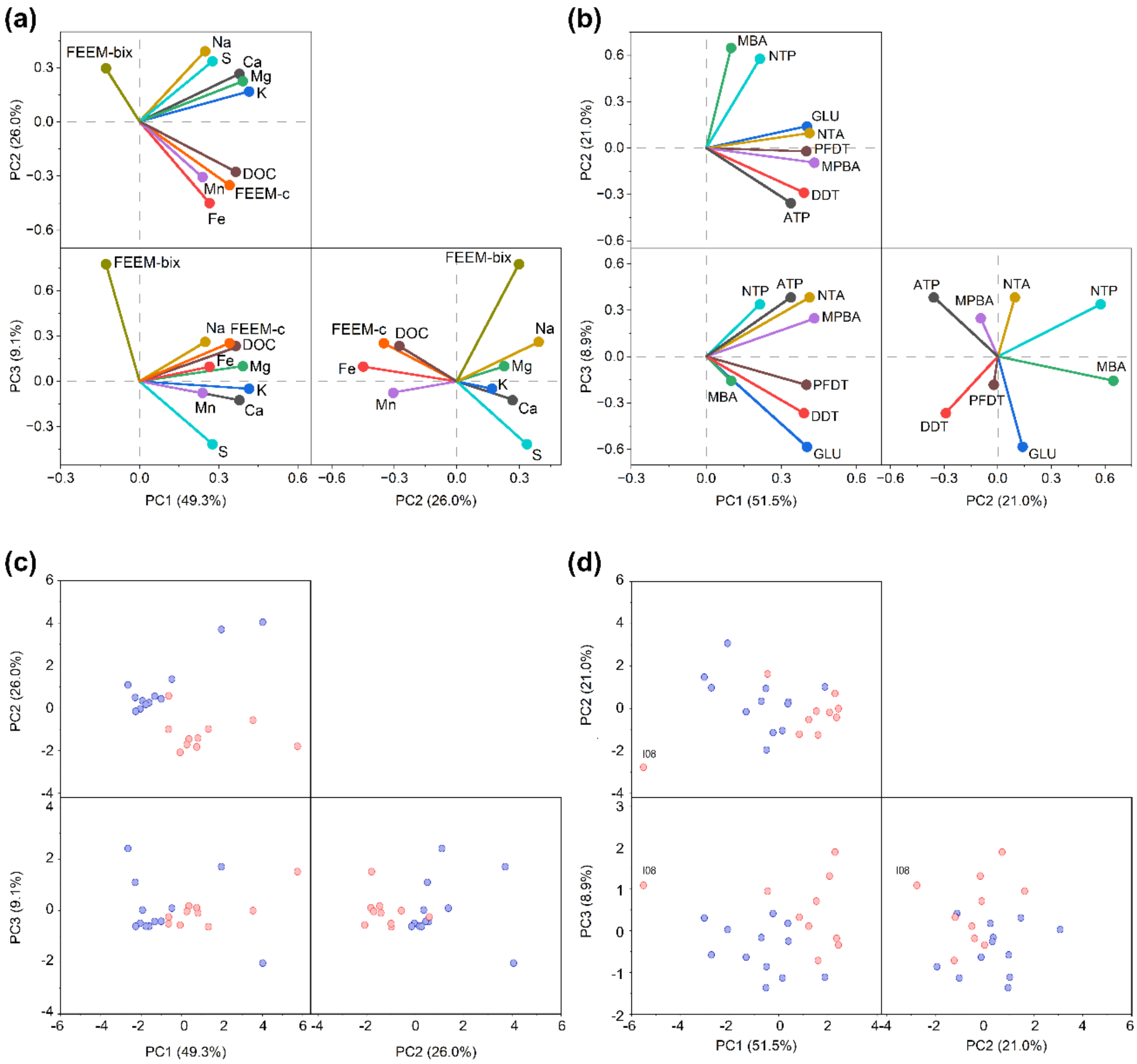

Figure S12. Eigenvectors plots for the first three components of the PCA of (a) the chemometric data and (b) the NTB sensor. PCA scatterplots of the first three PCs of the PCA of (c) the chemometric data and (d) the NTB sensor.

9. Linear discriminant analysis (LDA)

LDA with k-fold cross validation (k=5) was used on the NTB sensor dataset to estimate accuracy generate the ROC curves (JMP17 Software) using the same matrix layout as shown in **Table S4**.

Table S6a corresponds to the averaged classification matrix with bootstrapping (2500) and no cross-validation. To validate the sensor, we used k-fold cross-validation (k=5) on the second LDA. This results in 4/5 of the data being used to train the model and 1/5 being used to validate the model, iterated 25 times. **Table S6b** corresponds to the accuracy of classifying the data used to train the model (the 4/5), in the model, itself, and **Table S6c** corresponds to the accuracy of classifying the validation set (the 1/5), in the model.

Table S6. Classification matrices for the LDA grouped by INF and EFF. **(a)** Average classification from bootstrapping 2500 reps, seed = 456, no cross-validation. **(b,c)** Average of 25 iterations using k-fold, cross-validation (k=5) for the training set and validation set, respectively.

(a)

		Predicted	
		INF	EFF
Actual	INF	100%	0%
	EFF	0%	100%

(b)

		Predicted	
		INF	EFF
Actual	INF	90%	10%
	EFF	0%	100%

(c)

		Predicted	
		INF	EFF
Actual	INF	70%	30%
	EFF	8.3%	91.7%

10. References

1 O. Du Roure, C. Debiemme-Chouvy, J. Malthête and P. Silberzan, Functionalizing Surfaces with Nickel Ions for the Grafting of Proteins, *Langmuir*, 2003, **19**, 4138–4143.

2 S. K. Ghosh, S. Nath, S. Kundu, K. Esumi and T. Pal, Solvent and Ligand Effects on the Localized Surface Plasmon Resonance (LSPR) of Gold Colloids, *J. Phys. Chem. B*, 2004, **108**, 13963–13971.

3 J. C. Love, L. A. Estroff, J. K. Kriebel, R. G. Nuzzo and G. M. Whitesides, Self-Assembled Monolayers of Thiolates on Metals as a Form of Nanotechnology, *Chem. Rev.*, 2005, **105**, 1103–1170.

4 T. Liyanage, M. Nagaraju, M. Johnson, B. B. Muhoberac and R. Sardar, Reversible Tuning of the Plasmoelectric Effect in Noble Metal Nanostructures Through Manipulation of Organic Ligand Energy Levels, *Nano Lett.*, 2020, **20**, 192–200.

5 C. Goldmann, R. Lazzari, X. Paquez, C. Boissière, F. Ribot, C. Sanchez, C. Chanéac and D. Portehault, Charge Transfer at Hybrid Interfaces: Plasmonics of Aromatic Thiol-Capped Gold Nanoparticles, *ACS Nano*, 2015, **9**, 7572–7582.

6 Y. Xue, X. Li, H. Li and W. Zhang, Quantifying thiol–gold interactions towards the efficient strength control, *Nat Commun*, 2014, **5**, 4348.

7 H. Häkkinen, The gold–sulfur interface at the nanoscale, *Nature Chem*, 2012, **4**, 443–455.

8 Q. Zhang and H. Wang, Mechanistic Insights on Plasmon-Driven Photocatalytic Oxidative Coupling of Thiophenol Derivatives: Evidence for Steady-State Photoactivated Oxygen, *J. Phys. Chem. C*, 2018, **122**, 5686–5697.

9 H. Su, Y. Wang, Z. Yu, Y. Liu, X. Zhang, X. Wang, H. Sui, C. Sun and B. Zhao, Surface-enhanced Raman spectroscopy study on the structure changes of 4-Mercaptophenylboronic Acid under different pH conditions, *Spectrochimica Acta Part A: Molecular and Biomolecular Spectroscopy*, 2017, **185**, 336–342.

10 B. Mir-Simon, I. Reche-Perez, L. Guerrini, N. Pazos-Perez and R. A. Alvarez-Puebla, Universal One-Pot and Scalable Synthesis of SERS Encoded Nanoparticles, *Chem. Mater.*, 2015, **27**, 950–958.
